# Anti-Lipid IgG Antibodies Are Produced *via* Germinal Centers in a Murine Model Resembling Human Lupus

**DOI:** 10.3389/fimmu.2016.00396

**Published:** 2016-09-29

**Authors:** Carlos Wong-Baeza, Albany Reséndiz-Mora, Luis Donis-Maturano, Isabel Wong-Baeza, Luz Zárate-Neira, Juan Carlos Yam-Puc, Juana Calderón-Amador, Yolanda Medina, Carlos Wong, Isabel Baeza, Leopoldo Flores-Romo

**Affiliations:** ^1^ Department of Cell Biology, Center for Research and Advanced Studies, CINVESTAV-IPN, National Polytechnic Institute, Mexico City, Mexico; ^2^ Laboratorio de Biomembranas, Departamento de Bioquímica, Escuela Nacional de Ciencias Biológicas (ENCB), IPN, Ciudad de México, México; ^3^ Laboratorio de Inmunología Molecular II, Departamento de Inmunología, ENCB, IPN, Ciudad de México, México; ^4^ Laboratory of monoclonal antibodies, Institute of Epidemiological Diagnosis and Reference, Mexico City, Mexico

**Keywords:** non-bilayer phospholipid arrangements, systemic lupus erythematosus, anti-lipid IgG antibodies, antigen-specific cells, autoimmune disease

## Abstract

Anti-lipid IgG antibodies are produced in some mycobacterial infections and in certain autoimmune diseases [such as anti-phospholipid syndrome, systemic lupus erythematosus (SLE)]. However, few studies have addressed the B cell responses underlying the production of these immunoglobulins. Anti-lipid IgG antibodies are consistently found in a murine model resembling human lupus induced by chlorpromazine-stabilized non-bilayer phospholipid arrangements (NPA). NPA are transitory lipid associations found in the membranes of most cells; when NPA are stabilized they can become immunogenic and induce specific IgG antibodies, which appear to be involved in the development of the mouse model of lupus. Of note, anti-NPA antibodies are also detected in patients with SLE and leprosy. We used this model of lupus to investigate *in vivo* the cellular mechanisms that lead to the production of anti-lipid, class-switched IgG antibodies. In this murine lupus model, we found plasma cells (Gr1^−^, CD19^−^, CD138^+^) producing NPA-specific IgGs in the draining lymph nodes, the spleen, and the bone marrow. We also found a significant number of germinal center B cells (IgD^−^, CD19^+^, PNA^+^) specific for NPA in the draining lymph nodes and the spleen, and we identified *in situ* the presence of NPA in these germinal centers. By contrast, very few NPA-specific, extrafollicular reaction B cells (B220^+^, Blimp1^+^) were found. Moreover, when assessing the anti-NPA IgG antibodies produced during the experimental protocol, we found that the affinity of these antibodies progressively increased over time. Altogether, our data indicate that, in this murine model resembling human lupus, B cells produce anti-NPA IgG antibodies mainly via germinal centers.

## Introduction

**Figure 1:**
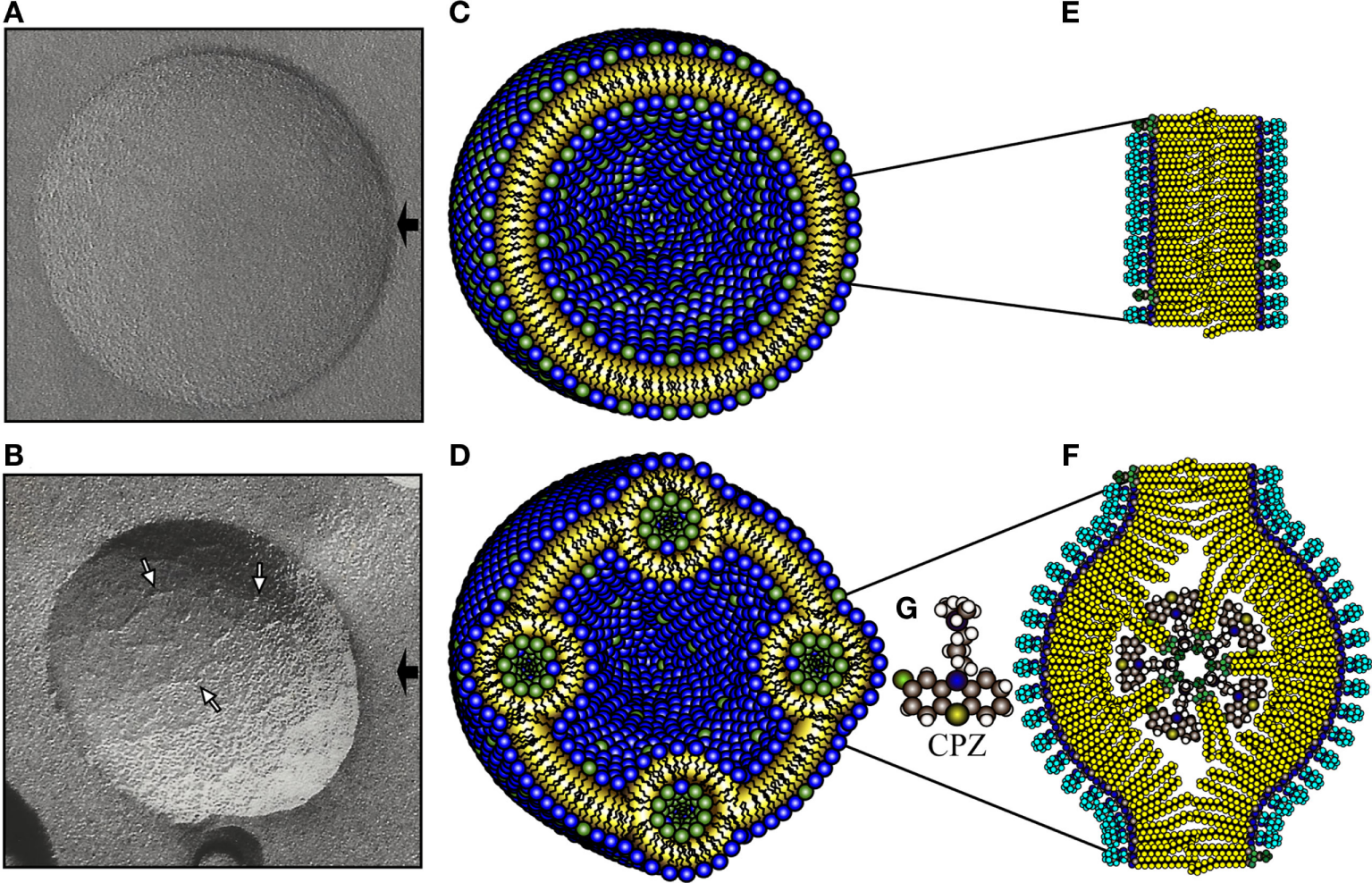
**NPA as detected by freeze-fracture electron microscopy, together with a schematic representation**. Freeze-fracture electron microscopy of liposomes made of l-α-phosphatidylcholine (PC)/L-α-phosphatidic acid (PA) (2:1 molar ratio) alone **(A)** or incubated with chlorpromazine (CPZ) 3 mM **(B)**. The black arrows indicate the shadow direction and the white arrows show NPA, either isolated or forming small strings. Schematic representation illustrates the molecular organization of the phospholipids in a smooth liposome without NPA **(C)** or bearing NPA **(D)**. The amplifications to the right depict the phospholipids in the bilayer arrangements **(E)** and in the NPA **(F)**. The bilayers in the NPA are mainly formed by PC, whose polar regions (blue color) are exposed on the zones of the lipid bilayer where the inverted micelle is inserted. The novel exposure of these polar regions of PC induces the production of antibodies against them. The inverted micelle is mainly formed by PA (polar regions in green color) together with CPZ (9). The molecular structure of CPZ is shown in **(G)**.

Anti-lipid IgG antibodies are produced during malaria, mycobacterial infections, autism spectrum disorders, and some autoimmune diseases, such as the anti-phospholipid syndrome and systemic lupus erythematosus (SLE) (1–4). Likewise, anti-lipid IgGs are consistently found in a mouse model of lupus that has several of the clinical and pathological features of SLE patients, including serum anti-nuclear, anti-histone, anti-coagulant and anti-cardiolipin antibodies, weight loss, glomerulonephritis, anorexia, and facial lesions resembling the malar rash of lupus patients (5). This model can be triggered either by the administration of chlorpromazine (CPZ), procainamide, or hydralazine, which induce stable non-bilayer phospholipid arrangements (NPA) on cell membranes, or by administering liposomes bearing NPA (L-NPA) elicited by these same agents or by cations, such as manganese, or even by injecting a NPA-specific monoclonal antibody (H308, which stabilizes NPA on murine cells). NPA are three-dimensional structures that can emerge when anionic phospholipids with a conical shape, such as phosphatidic acid (PA), phosphatidylserine, or cardiolipin, produced an intermediate form of the tubular hexagonal phase II or inverted micelle that is inserted in a bilayer of lipids (**Figure 1**) (6–8). NPA are transient lipid associations found on the membrane of most cells; when they are stabilized, they can become immunogenic and induce the production of specific antibodies that participate in the pathogenesis of this mouse model of lupus (5, 9); moreover, anti-NPA antibodies are found in patients with SLE and leprosy (5, 10). On the other hand, liposomes made of a lipid extract from *Mycobacterium smegmatis* elicit high titers of anti-lipid IgG antibodies, which are cross-reactive with lipid antigens from *Mycobacterium tuberculosis* (1). However, few studies have addressed the cellular reactions that lead to the production of these anti-lipid IgG antibodies.

In adaptive antibody responses to most protein antigens, activation and proliferation of B cells occur either in secondary follicles where B cells form germinal centers, or in extrafollicular foci (11–13). Germinal center B cells (IgD^−^, CD19^+^, PNA^+^) switch the antibody isotype and mutate the genes that encode their antigen receptors. These processes can change the antibody affinity and even the antibody specificity. The mutated cells that produce high-affinity antibodies are selected to become either plasma cells (Gr1^−^, CD19^−^, CD138^+^) or memory B cells, whereas cells that have lost affinity or acquired autoreactivity are typically eliminated (14, 15). Normally, CD4^+^ T (follicular) helper cells are critical for the germinal center formation and the subsequent B cell selection. Both processes involve engagement of at least CD40 on B cells by CD40-ligand on T cells, although there are reports describing the generation of high-affinity B cells in large germinal centers in the absence of T cells, or in absence of signaling through CD40 or CD28. However, in these latter cases, extensive cross-linking of the B cell receptors, and a high frequency of antigen-specific B cells of at least 1 in 1,000, is required (16). Extrafollicular reactions are responsible for the fast production of antibodies upon antigen encounter, and the extrafollicular B cells (B220^+^, Blimp1^+^) are typically found in the medullary cords of lymph nodes and in foci in the spleen red pulp. In some circumstances, the extrafollicular responses can be associated with immunoglobulin class switching but, at the most, with only low levels of hypermutation (11, 17). In our study, we used an established murine model resembling human lupus induced by the stabilization of NPA to investigate if the cellular mechanisms that lead to the production of anti-NPA IgG antibodies are through germinal centers or through extrafollicular reactions.

## Materials and Methods

### Preparation and Characterization of Liposomes and Liposomes Bearing NPA

Egg yolk l-α-PA, egg yolk l-α-phosphatidylcholine (PC) and CPZ were purchased from Sigma Aldrich (St. Louis, MO, USA). Liposomes were formed with the cylindrical phospholipid PC and the anionic and conical phospholipid PA in a molar ratio of 2:1. Nine micromoles of the phospholipid mixture were dissolved in 1 mL of diethyl ether, 330 μL of TS (10 mM Tris-HCl, 1 mM NaCl, pH 7) were added, and the mixture was sonicated three times (5-s sonication followed by 30 s resting period) in a Lab Supply G112SPI sonicator (Laboratory Supplies, Hicksville, NY, USA). The diethyl ether was then removed under a stream of oxygen-free dry nitrogen at reduced pressure, using a rotary evaporator at 37°C. TS was added to a final volume of 1 mL and the liposomes were filtered through MF-Millipore membranes (Billerica, MA, USA) with 0.45 μm pores. To induce the formation of NPA, liposomes in TS were incubated 30 min at 37°C in the presence of CPZ (3 mM) (18). To stain the liposomes, 100 μL of the red-fluorescent PKH26 dye (Sigma Aldrich) diluted 1:500 in diluent C (Sigma Aldrich) were added to 100 μL of smooth liposomes (SL) or L-NPA and incubated for 5 min at room temperature. To stop the staining, 200 μL of phosphate buffered saline (PBS) with 1.0% bovine serum albumin (BSA) (US Biologicals, Swampscott, MA, USA) were added, and then centrifuged at 268,000 × *g* for 15 min in the ultracentrifuge Optima TM MAX-XP (Beckman Coulter, Brea, CA, USA). The supernatant was discarded and the liposomes resuspended in PBS. All the final preparations of liposomes were negative for LPS contamination, as assessed by the gel clot LAL method (Charles River Endosafe, Charleston, SC, USA). The final preparations were evaluated by freeze-fracture electron microscopy, as reported before (9).

### Mouse Model of Lupus

To induce the lupus-like disease in mice, we used the protocol as described before (5, 9). Briefly, we used three groups (A, B, and C) of 6-week-old female pathogen-free BALB/c mice. Each group was divided into three subgroups of six mice each. Subgroup one received saline solution (SS), subgroup two SL and subgroup three liposomes bearing CPZ-induced NPA, on days 1 and 16 (50 μL, intrasplenic). Before the first injection, mice received intraperitoneally 100 μL of complete Freund’s adjuvant (CFA, Sigma Aldrich) diluted 1:2 with TS, and before the second injection, the mice received 100 μL of incomplete Freund’s adjuvant (Sigma Aldrich) diluted 1:2 with TS. Additionally, on days 2, 17, and 32, 50 μL of SS, SL, or liposomes bearing CPZ-induced NPA, were injected intradermally in the inguinal region of these mice, instead of the intraperitoneal region as was previously described (5). Blood was taken before and 10, 20, and 35 days after the first intra-splenic injection. Sera were heated at 56°C for 30 min to inactivate complement, and then frozen in aliquots at −70°C. Mice of groups A, B, and C were sacrificed on days 10, 20, and 35, respectively, after the first intra-splenic injection (Protocol illustrated in **Figure S1** in **Supplementary Material**).

The spleens and inguinal lymph nodes (ILN) of each mouse of the A, B, or C groups were extracted, sectioned and the cell suspensions obtained were placed in fluorescence-activated cell sorting (FACS) buffer solution containing PBS with 0.1% BSA and 0.01% sodium azide (Sigma Aldrich) for flow cytometry analysis. Bone marrows were extracted and placed also in FACS buffer solution for flow cytometry studies. All our protocols for animal care and use were reviewed and approved by the Bioethics Committee of our Institution following international rules (19).

### Confirmation of the Development of the Lupus-Like Disease in Mice in Response to Intradermal Injection of Liposomes Bearing NPA

A group of 10 mice was injected intradermally in the inguinal region once a week with liposomes bearing CPZ-induced NPA, and sacrificed 4 months after the first intra-splenic injection to confirm that these mice, which were injected intradermally instead of intraperitoneally, also developed the autoimmune disease resembling human lupus. Blood samples were taken every month, anti-NPA antibodies, as well as anti-histone and anti-cardiolipin antibodies, were measured in their sera, as reported before (5). A goat peroxidase-tagged anti-mouse antibody to IgG, IgM, and IgA isotypes was used to assess total antibodies to NPA; whereas goat peroxidase-tagged anti-mouse to total IgGs (Sigma Aldrich) diluted 1:2,000 was used as a secondary antibody in the ELISA to determine NPA-specific IgG antibodies only. Where indicated, L-NPA stained with the PKH26 red-fluorescent dye were used as antigens in the ELISA, in order to verify that NPA-fluorescent labeling did not modify the Ab recognition of NPAs.

### Identification of NPA-Specific B Cells and Plasma Cells by Flow Cytometry

Inguinal lymph nodes, spleens, and bone marrow were macerated in glass mortars and then filtered with a 75 μm cell strainer (Becton Dickinson, San Jose, CA, USA). The organs from two mice from the same subgroup were pooled together for analysis. Red blood cells from spleens were lysed and cell suspensions were resuspended in FACS buffer solution. Before staining, cells were incubated with Universal Blocking Reagent (Block Biogenex, San Ramón, CA, USA) diluted 1:10 with PBS for 10 min at 4°C and then washed. Plasma cells, germinal center, and extrafollicular B cells were labeled with the corresponding set of antibodies indicated below, and with L-NPA, which were previously stained with the lipophilic red-fluorescent dye PKH26. This latter procedure allowed us, therefore, to analyze the populations specific for the inducing antigen (NPA).

To evaluate NPA-specific germinal center B cells, cell suspensions were labeled with anti-IgD-PerCP/Cy5.5, anti-CD19-APC and PNA-FITC (Vector Laboratories, Burlingame, CA, USA) and 10 μL of the PKH26-stained liposomes for 30 min at 4°C and then washed and fixed with 1% paraformaldehyde (Sigma Aldrich).

Extrafollicular B cells were labeled with anti-B220-FITC, anti-CD5-AF647, and 10 μL of the PKH26-stained liposomes for 30 min at 4°C and then washed. Subsequently, 50 μL of Cytofix/Citoperm (Becton Dickinson) was added; and after 10 min at 4°C, the cells were washed with PermWash buffer (Becton Dickinson) 1×; an anti-Blimp1-biotinylated antibody (Abcam, Burlingame, CA, USA), followed by streptavidin coupled to Pacific Blue (Invitrogen, Carlsbad, CA, USA) diluted in 50 μL of PermWash buffer, was added, and the cells were incubated for 30 min at 4°C. The cells were washed a final time with PermWash buffer and fixed with 1% paraformaldehyde.

To evaluate NPA-specific plasma cells, cell suspensions were labeled first with anti-Gr1-PerCP, anti-CD19-APC, and with anti-CD138-Brilliant Violet 421 for 30 min at 4°C and then washed. Subsequently the procedure for intracellular labeling was as described: 50 μL of Cytofix/Citoperm was added; and after 10 min at 4°C, the cells were washed with PermWash buffer. Anti-IgG-FITC (Jackson Immunoresearch) diluted in 50 μL of PermWash buffer and 10 μL of the PKH26-stained liposomes were added, the cells were incubated for 30 min at 4°C, washed with PermWash buffer and fixed with 1% paraformaldehyde.

The samples were acquired in a BD LSR Fortessa cytometer (Becton Dickinson) and analyzed with FlowJo 10.0.6 (Tree Star, Inc., Ashland, OR, USA). Appropriate isotype controls were included in all sets of experiments. All antibodies were from BioLegend (San Diego, CA, USA) unless otherwise indicated.

### *In Situ* Identification of NPAs in Germinal Centers by Immunofluorescence

To identify NPAs in germinal centers, mice were sacrificed at day 35 after NPA inoculations and the ILNs were removed, embedded in Tissue-Tek (Sakura Finetek, Torrance, CA, USA), frozen, and stored at −70°C. Tissues were cut in 6-μm sections using a cryostat (Leica 1900 CM, Wetzlar, DE), sections were mounted on poly-l-Lysine-charged slides, fixed in 2% formaldehyde (Polysciences, Warrington, PA, USA) for 1 h at room temperature and washed in PBS containing 0.1% BSA. Tissue sections were treated with Universal Blocking Reagent (1:10) for 10 min at room temperature, and with mice sera diluted 1:10 in PBS for 10 min at room temperature. ILN sections were incubated overnight at 4°C with the following combination of antibodies: biotinylated H308 (NPA-specific) mouse monoclonal antibody and PNA-FITC, or with H308 and rat anti-IgD (Becton Dickinson) or with H308 and rat anti-B220 (Becton Dickinson). After further washes, slides were incubated 1 h at room temperature with anti-rat-AF488 (Thermo Fisher Scientific, Waltham, MA, USA) and washed three times. Subsequently, the slides were incubated for 15 min at room temperature with streptavidin-AF610 (Thermo Fisher Scientific) and washed. Finally, blue nuclear counter-staining was done using DAPI (Invitrogen) for 5 min at room temperature and the slides were washed. Slides were then mounted with DABCO (Sigma Aldrich) and examined with Image-Pro PLUS software in an Olympus BX51 microscope (Olympus Corporation, Hachioji, TY, JPN). Appropriate isotype controls were included in each experiment.

### Anti-NPA IgG Antibodies and Affinity Maturation during the Experimental Procedure

To determine whether the IgG antibodies to NPA underwent affinity maturation during the time course of the experimental protocol, we performed antigen-specific ELISAs for the anti-NPA IgG antibodies, both in the absence and in the presence of urea (Sigma Aldrich) 7M (20–22). ELISAs were performed as indicated above (5), but after adding the sera containing the anti-NPA IgG antibodies, urea 7M was added to each well for 10 min (secondary reagents were added after the corresponding washes). Under the astringent effects of urea, the antibodies with low affinities for antigen will detach more easily than the antibodies bound with higher affinities, and the result of this can be quantitated. Therefore, in this assay, the percentage of urea-resistant IgG anti-NPA antibodies can be calculated regarding the total IgG anti-NPA antibodies. The kinetic follow-up of this behavior during the immunization procedures will indicate whether affinity maturation of these antibodies has occurred. While low percentage indicates that most NPA-specific antibodies detached from antigen (low affinity), high percentage reveals that most antibodies remained attached to antigen despite the high-stringency urea wash (high affinity).

## Results

### Characterization of Liposomes Bearing CPZ-Induced NPA

Freeze-fracture electron microscopy of PC/PA (2:1) liposomes revealed vesicles with smooth surfaces (**Figure 1A**), as previously described (9, 18). The addition of 3 mM CPZ induced the formation of NPA (**Figure 1B**), which have an average thickness of 5 nm; these NPA are isolated or form small strings that do not modify the liposome size. PC/PA (2:1) liposomes bearing CPZ-induced NPA and stained with PKH26 dye showed an image similar to the liposomes bearing CPZ-induced NPA, which have not been PKH26-stained (data not shown). A schematic representation of SL, or L-NPA, is shown (**Figures 1C-G**).

### Mice Inoculated with Liposomes Bearing NPA Produce IgG Anti-NPA Antibodies

**Figure 2:**
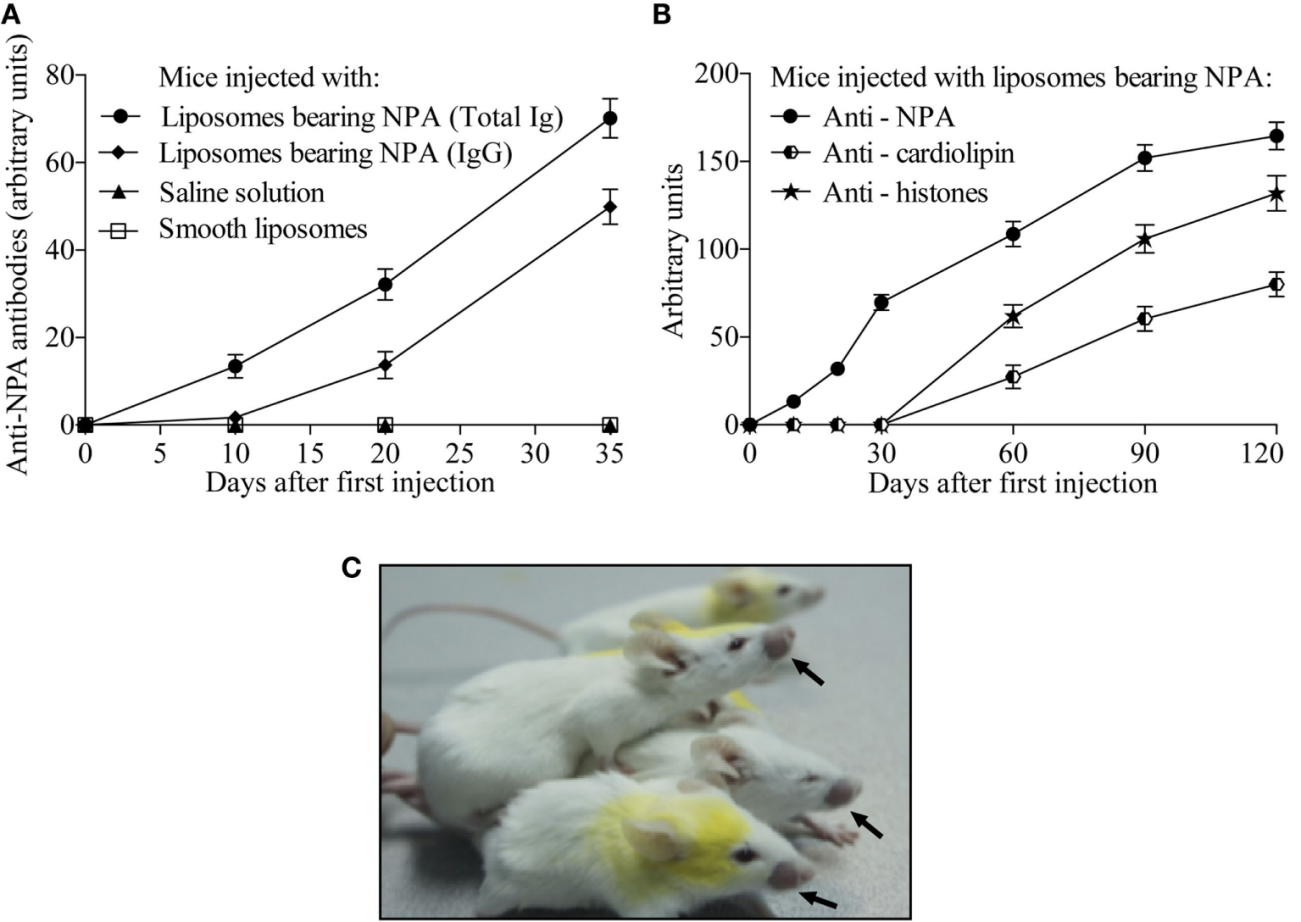
**Mice injected with liposomes bearing NPA produced IgG antibodies that specifically bind to NPA**. Sera of mice injected either with saline solution, with smooth liposomes, or with liposomes bearing NPA were assayed by ELISA at the indicated days using as antigen the liposomes bearing NPA. Goat anti-mouse antibody to the IgG, IgM and IgA isotypes was used to assess total antibodies to NPA; whereas goat anti-mouse to IgG was used to assess only the IgG specific to NPA **(A)**. Anti-NPA, anti-histones, and anti-cardiolipin antibodies were also measured by ELISA in the sera of mice injected intradermally with liposomes bearing NPA once a week for 4 months **(B)**; 60% of these mice developed facial lesions, as indicated by the arrows **(C)**.

Anti-NPA antibodies were readily detected in the sera of mice that received L-NPA intradermally; while these antibodies were not detected in mice that received only SL or SS. A proportion of the anti-NPA antibodies (13, 43, and 71% on days 10, 20, and 35 after first immunization, respectively) were of the IgG class (**Figure 2A**). In the mice that were injected intradermally in the inguinal region with L-NPA, anti-NPA antibodies were also detected since the 10th day after the first inoculation, and these antibodies increased progressively until the fourth month. Anti-histone and anti-cardiolipin antibodies were detected a month later than the anti-NPA antibodies, and they also increased progressively until the fourth month (**Figure 2B**), which is consistent with previous reports (5, 9). These mice also developed facial lesions on the second month after the first injection of L-NPA (**Figure 2C**).

### Plasma Cells That Produce IgGs Specific to NPA Are Found in Mice with the Lupus-Like Disease

**Figure 3:**
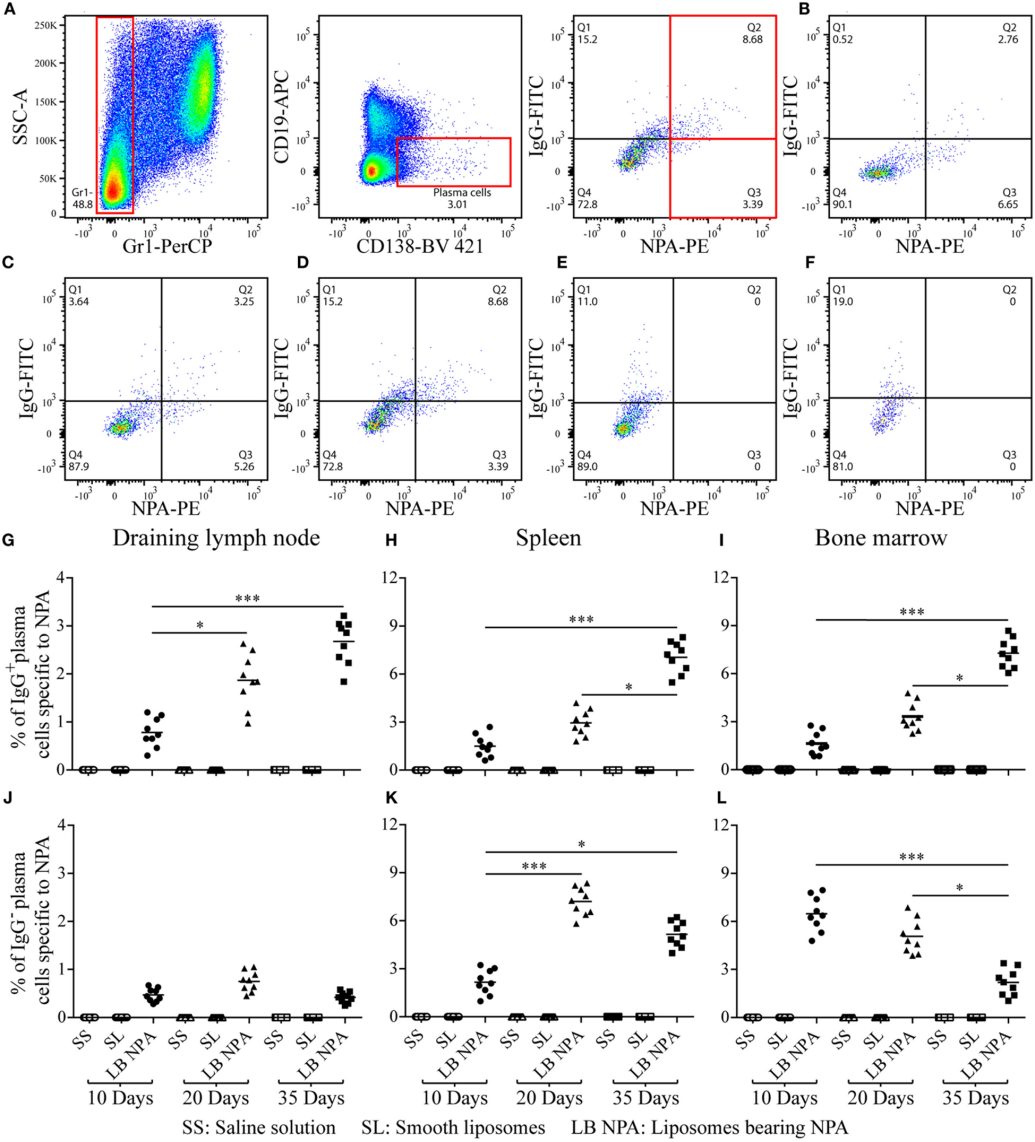
**NPA-specific plasma cells from mice inoculated with liposomes bearing NPA**. To identify NPA-specific plasma cells, cell suspensions from the draining lymph nodes, spleen, and bone marrow were co-labeled with antibodies to surface Gr1, CD19, and CD138 and intracellularly for IgG and with PKH26-stained liposomes bearing NPA (NPA-PE), and were then analyzed by flow cytometry. Gating strategy to identify plasma cells specific to NPA (Gr1^−^, CD19^−^, CD138^+^, NPA^+^) **(A)**. Region analysis shows the percentage of NPA-specific IgG^+^ and IgG^−^ plasma cells on days 10 **(B)** 20 **(C)**, and 35 **(D)** in the bone marrow of mice that had been inoculated with NPA-liposomes. Percentage of NPA-specific IgG^+^ and IgG^−^ plasma cells on day 35 in the spleens of mice inoculated with saline solution **(E)** or with smooth liposomes **(F)**. Percentage of IgG^+^ or IgG^−^ plasma cells specific to NPA in the draining lymph nodes **(G,J)**, spleen **(H,K)**, and bone marrow **(I,L)**. Kruskal–Wallis test with Dunn’s post-test were used for statistical analysis; significance was set at *P* < 0.05. Asterisks represent statistically significant differences between the indicated groups (*P < 0.05, ***P < 0.001). Each symbol represents pooled cells from two mice. SS, saline solution; SL, smooth liposomes; LB NPA, liposomes bearing NPA.

To investigate the *in vivo* cellular mechanisms that lead to the production of IgG anti-NPA antibodies in this mouse model of lupus, we used flow cytometry. Cell suspensions were labeled with antibodies and with L-NPA stained with the red-fluorescent lipophilic dye PKH26, in order to identify cell populations specific for the inducing NPA antigen. PKH26-stained liposomes were tested as antigens in an ELISA, and no significant differences in the anti-NPA antibody titers were found, compared to non-stained L-NPA (data not shown). Specific plasma cells for NPA (Gr1^−^, CD19^−^, CD138^+^, NPA^+^) were identified by flow cytometry (**Figures 3A–D**) in the draining lymph nodes, the spleen and the bone marrow of mice that received L-NPA; these NPA-specific plasma cells were not observed in mice that received either SL or SS (**Figures 3E,F**). The percentage of IgG^+^ NPA-specific plasma cells increased progressively, from 0.78, 1.5, and 1.63% on day 10 to 2.67, 7.04, and 7.27% on day 35 for the draining lymph nodes, the spleen and bone marrow, respectively (**Figures 3G–I**). Concomitantly, the percentage of IgG^−^ NPA-specific plasma cells decreased progressively from 0.75 and 7.2% on day 20 to 0.41 and 5.15% on day 35 for the draining lymph nodes and the spleen, respectively (**Figures 3J,K**); and from 6.48% on day 10 to 2.19% on day 35 for bone marrow (**Figure 3L**).

### NPA-Specific Germinal Center B Cells Are Found in Mice with the Lupus-Like Disease

**Figure 4:**
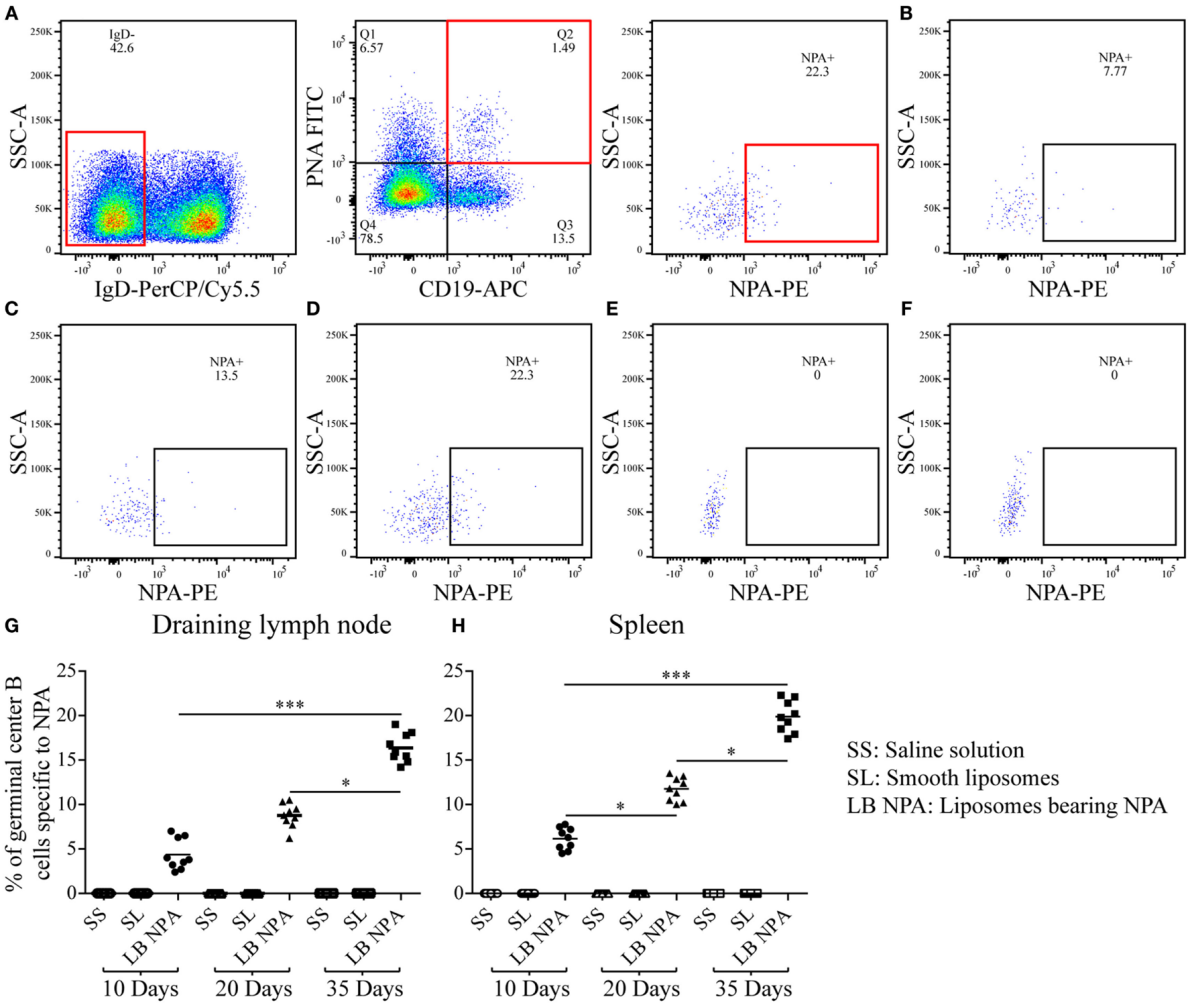
**Germinal center B cells specific to NPA from mice inoculated with liposomes bearing NPA**. In order to identify NPA-specific germinal center B cells, cell suspensions from the draining lymph nodes and spleens were co-labeled with red-fluorescent PKH26-stained liposomes bearing NPA (NPA-PE), with antibodies to surface IgD and CD19 and with fluorescent PNA and analyzed by flow cytometry. Gating strategy to identify NPA-specific germinal center B cells (IgD^−^, CD19^+^, PNA^+^, NPA^+^) **(A)**. Region analysis shows the percentage of NPA-specific germinal center B cells on days 10 **(B)**, 20 **(C)**, and 35 **(D)** in the spleens of mice that had been inoculated with NPA-liposomes. Percentage of NPA-specific germinal center B cells on day 35 in the spleens of mice inoculated with saline solution **(E)** or with smooth liposomes **(F)**. Percentages of germinal center B cells specific to NPA in the draining lymph nodes **(G)** and spleen **(H)**. Kruskal–Wallis test with Dunn’s post-test were used for statistical analysis; significance was set at *P* < 0.05. Asterisks represent statistically significant differences between the indicated groups (*P < 0.05, ***P < 0.001). Each symbol represents pooled cells from two mice. SS, saline solution; SL, smooth liposomes; LB NPA, liposomes bearing NPA.

Germinal center B cells specific for NPA (IgD^−^, CD19^+^, PNA^+^, NPA^+^) were identified by flow cytometry (**Figures 4A–D**) in the draining lymph nodes and the spleen of mice that received L-NPA; these germinal center B cells were not found in mice that received only SL or SS (**Figures 4E,F**). The percentage of NPA-specific germinal center B cells increased progressively from 4.37 and 6.15% on day 10 to 16.37 and 19.87% on day 35 for the draining lymph nodes and the spleen, respectively (**Figures 4G,H**).

### Few NPA-Specific Extrafollicular B Cells Are Found in Mice with the Lupus-Like Disease

**Figure 5:**
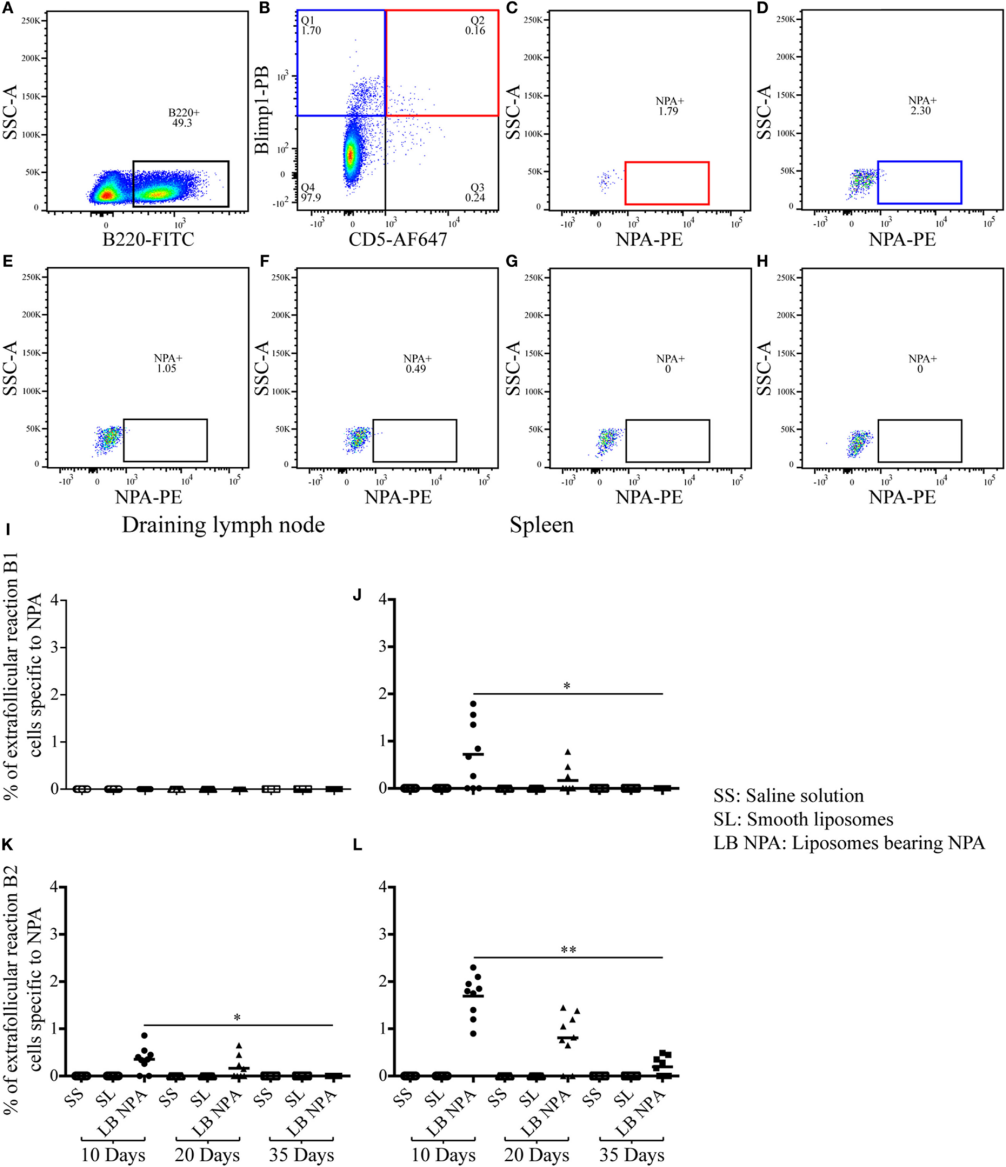
**Few extrafollicular reaction B cells specific to NPA were found in mice inoculated with liposomes bearing NPA**. To identify NPA-specific extrafollicular reaction B cells, cell suspensions from the draining lymph nodes and spleen were concomitantly labeled with red-fluorescent PKH26-stained liposomes bearing NPA (NPA-PE), with antibodies to surface B220 and CD5 and to intracellular Blimp1, and analyzed by flow cytometry on the indicated days. Gating strategy to identify extrafollicular reaction B1 (B220^+^, CD5^+^, Blimp1^+^, NPA^+^) **(A,B,C)** and B2 (B220^+^, CD5^−^, Blimp1^+^, NPA^+^) **(A,B,D)** cells specific to NPA. Region analysis shows the percentage of NPA-specific extrafollicular reaction B2 cells on days 10 **(D)**, 20 **(E)**, and 35 **(F)** in the spleens of mice that had been inoculated with NPA-liposomes. Percentage of NPA-specific extrafollicular reaction B2 cells on day 35 in the spleens of mice inoculated with saline solution **(G)** or with smooth liposomes **(H)**. Percentages of extrafollicular reaction B1 and B2 cells specific to NPA in the draining lymph node **(I,K)** and spleen **(J,L)**, respectively. Kruskal–Wallis test with Dunn’s post-test were used for statistical analysis; significance was set at *P* < 0.05. Asterisks represent statistically significant differences between the indicated groups (*P < 0.05, **P < 0.01). Each symbol represents pooled cells from two mice. SS, saline solution; SL, smooth liposomes; LB NPA, liposomes bearing NPA.

Few NPA-specific B1 (B220^+^, CD5^+^, Blimp1^+^, NPA^+^) and B2 (B220^+^, CD5^-^, Blimp1^+^, NPA^+^) cells that respond in an extrafollicular manner were identified by flow cytometry (**Figures 5A–F**) in the spleen of mice that received L-NPA; however only extrafollicular B2 cells specific to NPA were identified in the draining lymph nodes. These extrafollicular reaction B cells were not observed in mice that received only SL or SS (**Figures 5G,H**). The percentage of extrafollicular reaction B1 cells specific for NPA decreased progressively from 0.72% on day 10 to 0.0% on day 35 in the spleen (**Figure 5J**); while these cells were not detected in the draining lymph nodes of any of the groups examined (**Figure 5I**). The percentage of extrafollicular reaction B2 cells specific for NPA decreased progressively from 0.35 and 1.69% on day 10 to 0.0 and 0.19% on day 35 for the draining lymph nodes and the spleen, respectively (**Figures 5K,L**).

### NPAs Are Found in the Germinal Centers of Mice with the Lupus-Like Disease Induced by NPA-Immunizations

**Figure 6:**
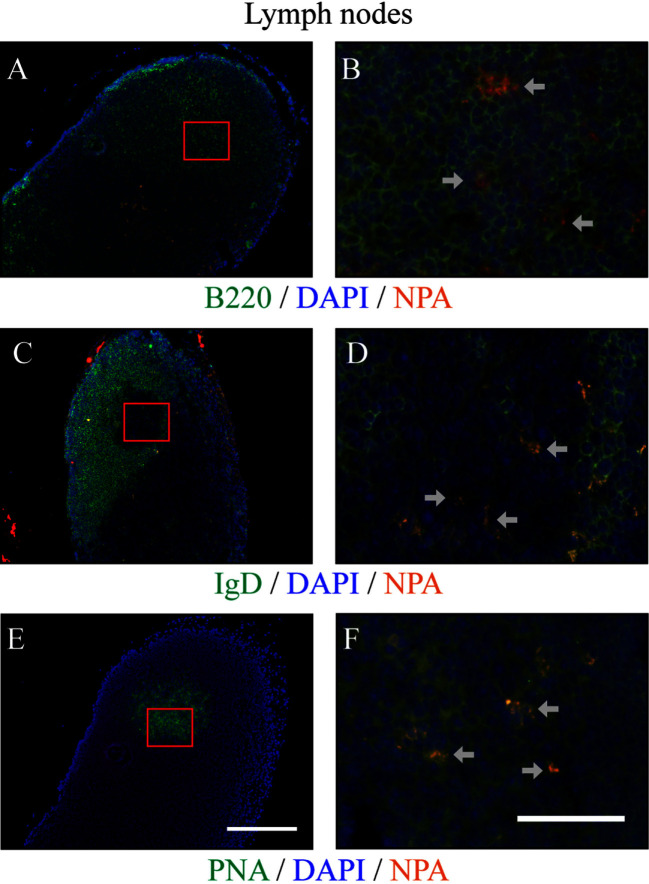
**NPAs can be found in germinal centers from draining lymph nodes of mice with lupus-like disease induced by NPA-immunizations**. The NPAs (identified with the monoclonal antibody H308, red fluorescence) were assessed in a series of double stainings with H308 and anti-B220 (green fluorescence) **(A,B)**, or with H308 and anti-IgD (green fluorescence) **(C,D)**, or with H308 and PNA (green fluorescence) **(E,F)**, in serial tissue sections from the draining inguinal lymph nodes of mice that developed the lupus-like disease. B cell follicles can be identified *in situ* with B220 [**(A,B)**, green fluorescence]. A negative IgD area [**(C,D)**, green fluorescence] inside B cell follicles indicates the germinal center; and this same IgD-negative zone corresponds to the PNA-positive labeling in the contiguous section [**(E,F)**, green fluorescence]. Nuclei counter-staining was done with DAPI (blue fluorescence). Germinal centers were distributed along the tissues and NPA staining revealed the positive cells in germinal centers (arrows). Representative pictures of three independent experiments are shown. For each marker, the images to the right (40×, scale bar = 50 μm) correspond to the red squares indicated on the images to the left (10×, scale bar = 200 μm). Co-localization of a given marker (green fluorescence) with NPA (red fluorescence) appears as orange-yellow. Images with individual colors are shown in **Figure S2** in **Supplementary Material**.

Using the NPA-specific monoclonal antibody H308, NPAs were identified by immunofluorescence in the germinal centers of the draining lymph nodes 35 days after mice received L-NPA. B220 delineates the B cell follicles (**Figures 6A,B**) and an IgD-negative area inside B cell follicles indicates the germinal centers (**Figures 6C,D**). These IgD-negative areas are positive for PNA (**Figures 6E,F**).

### The Affinity of the Anti-NPA IgG Antibodies Progressively Increases over Time

**Figure 7:**
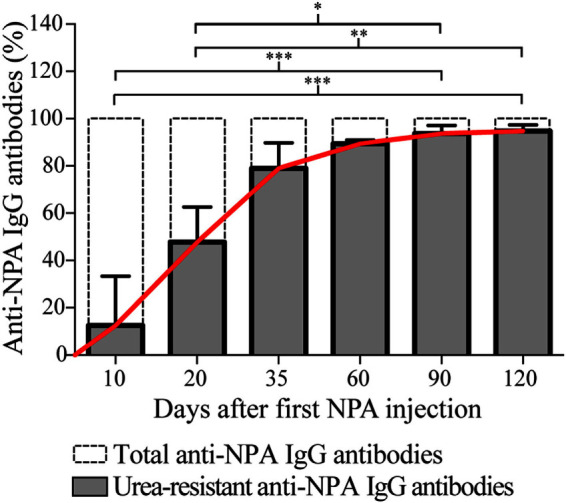
**The affinity of the anti-NPA IgG antibodies increases with time after immunization**. Sera of mice injected at different time points with liposomes bearing NPA were assayed by ELISA on the indicated days, using liposomes bearing NPA as antigen, and goat anti-mouse IgG as secondary antibody. After incubation of the anti-NPA antibodies, urea 7M was added to each well for 10 min (secondary reagents were added after this). At each time point, the percentage of urea-resistant anti-NPA IgG antibodies (high affinity, shown in gray bars) and the total anti-NPA IgG antibodies (indicated with dotted bars) are represented. The progressive increase in the affinity of the IgG anti-NPA antibodies is indicated with the continuous red line; at late time points (day 35 onwards) most antibodies display high affinities. Percentages of urea-resistant antibodies at the indicated times were analyzed with Kruskal–Wallis test with Dunn’s post-test; significance was set at *P* < 0.05. Asterisks represent statistically significant differences between the indicated groups (*P < 0.05, **P < 0.01, ***P < 0.001).

To determine if affinity maturation, a hallmark of the antibodies produced via germinal center reactions, occurs during the course of the NPA-immunizations, we performed antigen-specific ELISAs for the anti-NPA IgG antibodies, both in the absence and in the presence of urea 7M. This is a well-established assay (20–22) used to test antibody affinities. Lower antibody affinities correlate with increased antibody decoupling from antigen under the astringent effects of urea. Conversely, higher antibody affinities lead to an increased proportion of antibodies that resist the effects of urea and remain bound to antigen. Using this assay, we obtained at each time point the percentage of urea-resistant (high affinity) anti-NPA IgG antibodies, in relation to the total anti-NPA IgG antibodies. As illustrated in **Figure 7**, the proportion of urea-resistant antibodies was much lower at early time points (10 and 20 days post-NPA-injection) than at later time points (35, 60, 90, and 120 days post-NPA injection), which indicates that affinity has increased with time. In fact, at days 35, 60, 90, and 120 post-immunization, most anti-NPA IgG antibodies showed high affinities, revealing the maturation of affinity.

## Discussion

Compared to proteins, lipids are deemed rather poorly immunogenic molecules (3). However, anti-lipid antibodies have been found in patients with infectious diseases, such as leprosy and tuberculosis (1, 10), and in patients with autoimmune diseases such as SLE (4); these antibodies are not only low-affinity IgM, but also high-affinity class-switched IgG antibodies. The cellular mechanisms that lead to the production of these IgM and IgG anti-lipid antibodies have not been elucidated and are poorly studied. To investigate these mechanisms, we assessed a mouse model of an autoimmune disease resembling human lupus, which develops in response to the administration of NPA (9). NPA are transient phospholipid molecular arrangements, different from the bilayer formed by lipids from cell membranes (8). These phospholipid associations are observed by electron microscopy as protuberances on the lipid matrix of liposomes (artificial membrane models). When lipids are associated in stable NPA, they become more immunogenic and induce the production of specific antibodies (5, 9). Here, we report for the first time that, in mice that developed an autoimmune disease resembling human lupus in response to the administration of NPA, B cells reacted mainly through the germinal centers to produce anti-NPA IgG antibodies.

In NPA, the polar heads of the phospholipids that form the outer region of this lipid structure are separated from each other, so they are exposed as new antigens; however, since NPA are transient structures, usually no immune response is elicited (5). Of note, when NPA are stabilized by drugs that induce lupus-like diseases in humans (23), such as CPZ, promazine, procainamide, and hydralazine, an immune response is generated with the production of anti-NPA antibodies. Interestingly, in mice, these same drugs trigger the development of an autoimmune disease similar to human lupus. We have already demonstrated, with hapten inhibition studies, that the epitope recognized by these antibodies is glycerolphosphorylcholine, the polar region of PC, which is the most abundant phospholipid in animal cell membranes (9).

In previous studies, we found that a high percentage (60%) of the anti-NPA antibodies that are found in the mice with the lupus-like disease are of the IgG class. To study the mechanisms that lead to the production of these anti-NPA switched IgG antibodies, we inoculated NPA to mice by intra-splenic injection (as we had done it before) (5, 9), but changed the administration of subsequent NPA boosting doses from intraperitoneal to intradermal injection in the inguinal region, so that we could monitor the (systemic) immune response in the spleen and in the draining (regional) lymph node. The mice that received NPA by the intradermal route also generated anti-NPA antibodies, and 45% of these antibodies were IgG. These mice produced anti-cardiolipin and anti-histone antibodies 1 month after the appearance of the anti-NPA antibodies, and 60% of the mice had malar rash. These results indicate that these mice had an autoimmune disease with very much the same characteristics to the disease that develops in response to the administration of NPA by the intraperitoneal route, as we previously reported (5, 9).

During the production of class-switched IgG antibodies against most protein antigens, B cells respond *in vivo* mainly via the germinal center or (to a lesser degree) the extrafollicular reaction, and subsequently differentiate into plasmablasts and plasma cells that produce IgG antibodies (11, 14, 24). To identify the specific cells producing the anti-NPA antibodies, liposomes with or without NPA were stained with the lipophilic (red fluorescent) dye PKH26. This staining did not affect the structure of the NPA in the liposomes, because L-NPA and stained with PKH26 were equally recognized by anti-NPA mouse sera or even by a monoclonal anti-NPA antibody, while control liposomes without NPA but equally stained with PKH26 were not recognized by these anti-NPA antibodies. Therefore, L-NPA and stained with PKH26 were used to identify anti-NPA antibody-producing cells by flow cytometry. With this strategy, we found plasma cells (Gr1^−^, CD19^−^, CD138^+^, NPA^+^) that produce anti-NPA antibodies in the secondary lymphoid organs (draining lymph node and spleen) of mice with the lupus-like disease. We also found plasma cells in the bone marrow, which is the organ to which these cells can migrate after their differentiation in the secondary lymphoid organs (25). To avoid false-positive results, granulocytes (Gr1^+^), which can uptake most types of small particles and molecules, including antibodies, were excluded from the flow cytometry analysis.

The percentage of plasma cells that produce anti-NPA IgG antibodies increased gradually in the three organs analyzed (lymph nodes, spleen, and bone marrow), while the percentage of IgG-negative NPA-specific plasma cells decreased in these same organs at 10, 20, and 35 days after the first administration of NPA. This plasma-cell response is similar to that described for protein antigens (26). A larger number of plasma cells specific to NPA was found in the spleen, compared to the draining lymph node, and this may be attributed to the populations of B cells (B1 cells and marginal zone B cells) that are present in the spleen, but not in the lymph nodes, and which could generate such plasma cells (25).

After identifying the specific plasma cells producing anti-NPA IgG antibodies, we investigated the route by which B cells responded, and found that the reaction of anti-NPA B cells was mainly via the germinal centers. Germinal centers provide the microenvironmental requirements for the interactions between follicular T and B cells that lead to the generation of high-affinity IgG antibodies against protein antigens (12). IgG antibodies against several lipids (lathosterol, 7-α-hydroxycholesterol, cardiolipin, and oxidized PC) have been found in SLE patients (27), and IgG antibodies against long-chain saturated fatty acids have been found in the sera of patients with type 2 diabetes (28). IgM and IgG anti-cholesterol antibodies have been detected even in healthy individuals, as well as in patients with atherosclerosis and viral infections (29–31). However, the cellular mechanisms that lead to the production of these anti-lipid antibodies are still poorly understood. The main cells providing help to B cells for the production of anti-lipid antibodies seem to be NKT cells rather than CD4 T cells. For instance, NKT cells, not conventional CD4 T cells, provide cognate help to B cells specific for 4-hydroxy-3-nitrophenyl-α-galactosyl-ceramide (NP-αGalCer), which produce anti-NP IgG antibodies (32). It has been shown that invariant-chain NKT cells (iNKT cells) can cooperate with B cells and induce the rapid formation of germinal centers to glycolipid antigens (33). iNKT cells can recognize synthetic, self- and microbial glycolipids, as well as self-phospholipid antigens presented by CD1d (34); and in patients with SLE it has been shown that iNKT cells promote the production of IgG antibodies and auto-antibodies (35). In fact, iNKT cells can adopt a phenotype that corresponds to that of follicular T helper cells, with high expression of CXCR5, PD-1, and Bcl-6, and the secretion of IL-4, IFN-γ, IL-21, and BAFF. These iNKT cells can be found in germinal centers and they provide help to B cells in a CD40–CD40L-dependent manner, which leads to the production of IgG antibodies (33, 36, 37). It is, thus, conceivable that iNKT cells might be cooperating with B cells for the production of anti-NPA IgG antibodies (38), but this needs to be demonstrated.

The percentage of germinal center B cells (IgD^−^, CD19^+^, PNA^+^, NPA^+^) specific to NPA was higher in the lymph nodes and the spleen than the percentage of extrafollicular reaction B2 cells (CD5^−^, B220^+^, Blimp1^+^, NPA^+^), especially on day 35 after the first immunization. We also found the antigen (NPA) inside the germinal centers of the draining lymph nodes of mice that developed the lupus-like disease. Extrafollicular reaction B1 cells (CD5^+^, B220^+^, Blimp1^+^, NPA^+^) were not detected in the draining lymph node (where they are usually absent), and very few were detected in the spleen, where they decreased until they became undetectable by day 35. In general, this pattern regarding the percentage of germinal center B cells and extrafollicular reaction B1 and B2 cells that reacted against NPA is similar to that described for protein antigens. Affinity maturation is a crucial process during antibody responses and occurs mainly during germinal center reactions; thus, the fact that we detected that the affinity of these anti-NPA IgG antibodies progressively augmented with time after immunization strongly supports the notion that these antibodies were produced via germinal centers.

The production of antibodies by plasma cells is a critical process to maintain human health as it provides immunity after the exposure to a pathogen and mediates the protective effects of most vaccines. While the B cell-differentiation process to generate plasma cells that produce antibodies to protein antigens has been extensively studied, reports regarding these events in the case of lipid antigens are scarce. However, these same anti-lipid antibodies-producing cells might be involved in clinically relevant diseases, such as autoimmunity and infection. It is crucial, therefore, to understand the mechanisms of B cell differentiation to plasma cell, particularly as it relates to the generation of class-switched IgG antibodies, not only in response to protein antigens but also to lipid antigens, which might be involved in clinically relevant autoimmune and infectious diseases.

## Ethics Statement

The study was approved by the Comité de Bioetica de la Escuela Nacional de Ciencias Biologicas (CEI-ENCB-025/2014) and the Comité de Bioetica del CINVESTAV (CD-2013-01).

## Author Contributions

Conceived and designed the experiments: CW-B, IB, and LF-R. Performed the experiments and the acquisition of data: CW-B, AR-M, LD-M, LZ-N, JY-P, JC-A, and YM. Analyzed the data, interpretation, and discussion: CW-B, IW-B, CW, IB, and LF-R. Wrote and revised the paper: CW-B, IW-B, IB, and LF-R. Final approval of the version to be published: LF-R.

## Conflict of Interest Statement

The authors declare that the research was conducted in the absence of any commercial or financial relationships that could be construed as a potential conflict of interest. CW and IB are listed as authors in the following patents: (1) US Patent 6,777,193, Methods for diagnostic and/or treatment of anti-phospholipid antibodies-related diseases and devices, and (2) US Patent 7,867,723, Methods for anti-phospholipid syndrome.

## Supplementary Material

Figure S1
**Three groups (A, B, and C) of adult mice were divided into three subgroups of six mice each**. One subgroup received saline solution (SS), the other smooth liposomes (SL), and the last one liposomes bearing NPA (L-NPA). On day 1, group A received complete Freund’s adjuvant (CFA) (intraperitoneally, i.p.) and SS, SL, or L-NPA (intrasplenic, i.s.); on day 2, SS, SL, or L-NPA (intradermally, i.d.) and sacrificed on day 10. On day 16, group B received the same as group A plus incomplete Freund’s adjuvant (ICFA, i.p.) and SS, SL, or L-NPA (i.s.); on day 17, SS, SL, or L-NPA (i.d.) and sacrificed on day 20. Finally, group C received the same as groups A and B plus SS, SL, or L-NPA (i.d.) on day 32 and sacrificed on day 35. Samples were taken before immunization and on the days of sacrifice.

Figure S2
**Individual and merged images of labeled tissue sections from draining lymph nodes**. Individual images of the draining lymph nodes from mice with lupus-like disease induced by NPA-immunizations were taken with the Olympus BX51 microscope; green fluorescence for B220 **(A)**, IgD **(E)**, and PNA **(I)**; blue fluorescence for nuclei counter-staining (DAPI) **(B,F,J)** and red fluorescence for NPAs **(C,G,K)**. Merged images of B220/DAPI/PNA **(D)**, IgD/DAPI/NPA **(H)**, and PNA/DAPI/NPA **(L)**. Images were merged with Image-Pro PLUS software.
