# Corrigendum: Anti-Lipid IgG Antibodies Are Produced *via* Germinal Centers in a Murine Model Resembling Human Lupus

**DOI:** 10.3389/fimmu.2017.00440

**Published:** 2017-04-12

**Authors:** Carlos Wong-Baeza, Albany Reséndiz-Mora, Luis Donis-Maturano, Isabel Wong-Baeza, Luz Zárate-Neira, Juan Carlos Yam-Puc, Juana Calderón-Amador, Yolanda Medina, Carlos Wong, Isabel Baeza, Leopoldo Flores-Romo

**Affiliations:** ^1^Department of Cell Biology, Center for Research and Advanced Studies, CINVESTAV-IPN, Instituto Politécnico Nacional (IPN), Mexico City, Mexico; ^2^Laboratorio de Biomembranas, Departamento de Bioquímica, Escuela Nacional de Ciencias Biológicas (ENCB), Instituto Politécnico Nacional (IPN), Mexico City, Mexico; ^3^Laboratorio de Inmunología Molecular II, Departamento de Inmunología, ENCB, Instituto Politécnico Nacional (IPN), Mexico City, Mexico; ^4^Laboratory of Monoclonal Antibodies, Institute of Epidemiological Diagnosis and Reference, Mexico City, Mexico

**Keywords:** non-bilayer phospholipid arrangements, systemic lupus erythematosus, anti-lipid IgG antibodies, antigen-specific cells, autoimmune disease

In the original article, the name of our Institution (National Polytechnic Institute, IPN) should have been written in full and in Spanish, as Instituto Politécnico Nacional (IPN), in the author’s affiliations section. The authors apologize for this oversight. This error does not change the scientific conclusions of the article in any way.

## Conflict of Interest Statement

The authors declare that the research was conducted in the absence of any commercial or financial relationships that could be construed as a potential conflict of interest.

