# Correction: Anti-lipid IgG antibodies are produced via germinal centers in a murine model resembling human lupus

**DOI:** 10.3389/fimmu.2025.1711763

**Published:** 2026-03-27

**Authors:** Carlos Wong-Baeza, Albany Reséndiz-Mora, Luis Donis-Maturano, Isabel Wong-Baeza, Luz Zárate-Neira, Juan Carlos Yam-Puc, Juana Calderón-Amador, Yolanda Medina, Carlos Wong, Isabel Baeza, Leopoldo Flores-Romo

**Affiliations:** 1 Department of Cell Biology, Center for Research and Advanced Studies, CINVESTAV-IPN, National Polytechnic Institute, Mexico City, Mexico; 2 Laboratorio de Biomembranas, Departamento de Bioquímica, Escuela Nacional de Ciencias Biológicas (ENCB), IPN, Ciudad de México, Mexico; 3 Laboratorio de Inmunología Molecular II, Departamento de Inmunología, ENCB, IPN, Ciudad de México, Mexico; 4 Laboratory of monoclonal antibodies, Institute of Epidemiological Diagnosis and Reference, Mexico City, Mexico

**Keywords:** non-bilayer phospholipid arrangements, systemic lupus erythematosus, anti-lipid IgG antibodies, antigen-specific cells, autoimmune disease

There was a mistake in **Figure 3** as published. We used the same image in panels 3A and 3D to show the gating strategy (panel 3A) and the experimental result (panel 3D).

Therefore, we deleted panel 3D and updated the letters on the remaining panels. The corrected **Figure 3** appears below.

**Figure 3:**
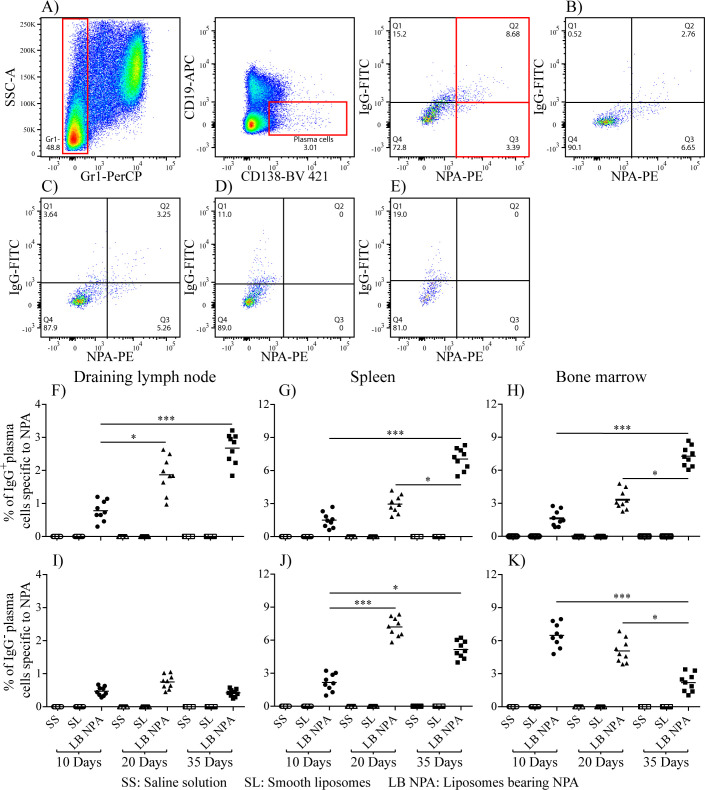
Region analysis shows the percentage of NPA-specific IgG+ and IgG- plasma cells on days 10 **(B)** 20 **(C)** and 35 **(A)** in the bone marrow of mice that had been inoculated with NPA-liposomes. Percentage of NPA-specific IgG+ and IgG- plasma cells on day 35 in the spleens of mice inoculated with saline solution **(D)** or with smooth liposomes **(E)**. Percentage of IgG+ or IgG- plasma cells specific to NPA in the draining lymph nodes **(F, I)**, spleen **(G, J)** and bone marrow **(H, K)**.

There was a mistake in the caption of **Figure 3** as published. We used the same image to show the gating strategy (panel 3A) and the experimental result (panel 3D). The caption of **Figure 3** with the mistake appears below:

“Region analysis shows the percentage of NPA-specific IgG^+^ and IgG^-^ plasma cells on days 10 (B) 20 (C) and 35 (D) in the bone marrow of mice that had been inoculated with NPA-liposomes. Percentage of NPA-specific IgG^+^ and IgG^-^ plasma cells on day 35 in the spleens of mice inoculated with saline solution (E) or with smooth liposomes (F). Percentage of IgG^+^ or IgG^-^ plasma cells specific to NPA in the draining lymph nodes (G, J), spleen (H, K) and bone marrow (I, L).”

Therefore, we deleted panel 3D, referred to panel 3A when describing the experimental result, and updated the letters on the remaining panels. The corrected caption of **Figure 3** appears below.

“Region analysis shows the percentage of NPA-specific IgG^+^ and IgG^-^ plasma cells on days 10 (B) 20 (C) and 35 (A) in the bone marrow of mice that had been inoculated with NPA-liposomes. Percentage of NPA-specific IgG^+^ and IgG^-^ plasma cells on day 35 in the spleens of mice inoculated with saline solution (D) or with smooth liposomes (E). Percentage of IgG^+^ or IgG^-^ plasma cells specific to NPA in the draining lymph nodes (F, I), spleen (G, J) and bone marrow (H, K).”

We used the same image to show the gating strategy (panel 3A) and the experimental result (panel 3D). The paragraph with the mistake appears below:

“Specific plasma cells for NPA (Gr1^-^, CD19^-^, CD138^+^, NPA^+^) were identified by flow cytometry (**Figures 3A-D**) in the draining lymph nodes, the spleen and the bone marrow of mice that received liposomes bearing NPA; these NPA-specific plasma cells were not observed in mice that received either smooth liposomes or saline solution (**Figures 3E, F**). The percentage of IgG^+^ NPA-specific plasma cells increased progressively, from 0.78%, 1.5% and 1.63% on day 10 to 2.67%, 7.04% and 7.27% on day 35 for the draining lymph nodes, the spleen and bone marrow, respectively (**Figures 3G-I**). Concomitantly, the percentage of IgG^-^ NPA-specific plasma cells decreased progressively from 0.75% and 7.2% on day 20 to 0.41% and 5.15% on day 35 for the draining lymph nodes and the spleen, respectively (**Figures 3J, K**); and from 6.48% on day 10 to 2.19% on day 35 for bone marrow (**Figure 3L**).”

Therefore, we deleted panel 3D, referred to panel 3A when describing the experimental result, and updated the letters on the remaining panels. A correction has been made to the section **Results** Section, subsection *Plasma cells that produce IgGs specific to NPA are found in mice with the lupus-like disease*, Paragraph 3:

“Specific plasma cells for NPA (Gr1^-^, CD19^-^, CD138^+^, NPA^+^) were identified by flow cytometry (**Figures 3A**–**C**) in the draining lymph nodes, the spleen and the bone marrow of mice that received liposomes bearing NPA; these NPA-specific plasma cells were not observed in mice that received either smooth liposomes or saline solution (**Figures 3D**, **E**). The percentage of IgG^+^ NPA-specific plasma cells increased progressively, from 0.78%, 1.5% and 1.63% on day 10 to 2.67%, 7.04% and 7.27% on day 35 for the draining lymph nodes, the spleen and bone marrow, respectively (**Figures 3F**–**H**). Concomitantly, the percentage of IgG^-^ NPA-specific plasma cells decreased progressively from 0.75% and 7.2% on day 20 to 0.41% and 5.15% on day 35 for the draining lymph nodes and the spleen, respectively (**Figures 3I**, **J**); and from 6.48% on day 10 to 2.19% on day 35 for bone marrow (**Figure 3K**).”

There was a mistake in **Figure 4** as published. We used the same image in panels 4A and 4D to show the gating strategy (panel 4A) and the experimental result (panel 4D).

Therefore, we deleted panel 4D and updated the letters on the remaining panels. The corrected **Figure 4** appears below.

**Figure 4:**
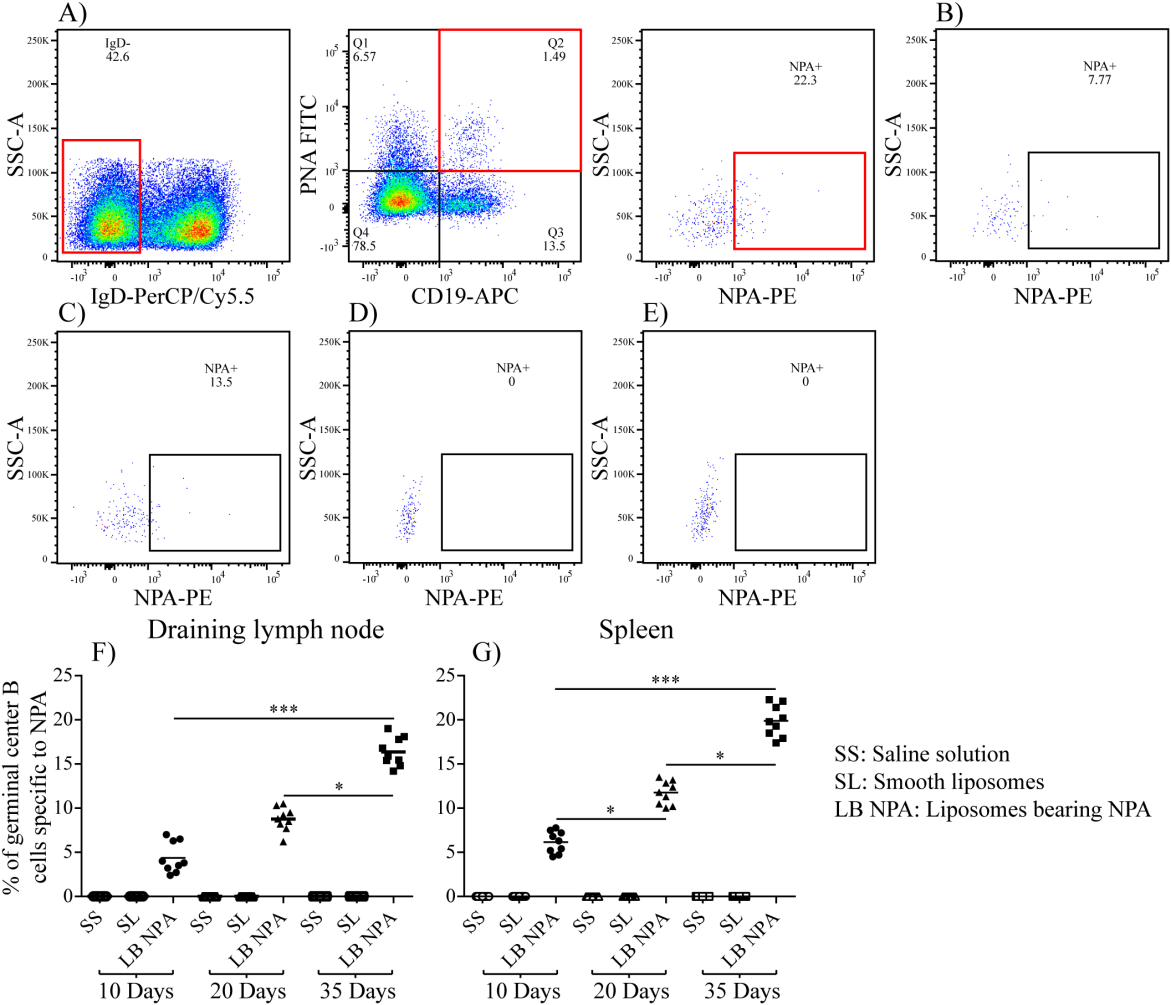
Region analysis shows the percentage of NPA-specific germinal center B cells on days 10 **(B)**, 20 **(C)** and 35 **(A)** in the spleens of mice that had been inoculated with NPA-liposomes. Percentage of NPA-specific germinal center B cells on day 35 in the spleens of mice inoculated with saline solution **(D)** or with smooth liposomes **(E)**. Percentages of germinal center B cells specific to NPA in the draining lymph nodes **(F)** and spleen **(G)**.

There was a mistake in the caption of **Figure 4** as published. We used the same image to show the gating strategy (panel 4A) and the experimental result (panel 4D). The caption of **Figure 4** with the mistake appears below:

“Region analysis shows the percentage of NPA-specific germinal center B cells on days 10 (B), 20 (C) and 35 (D) in the spleens of mice that had been inoculated with NPA-liposomes. Percentage of NPA-specific germinal center B cells on day 35 in the spleens of mice inoculated with saline solution (E) or with smooth liposomes (F). Percentages of germinal center B cells specific to NPA in the draining lymph nodes (G) and spleen (H).”

Therefore, we deleted panel 4D, referred to panel 4A when describing the experimental result, and updated the letters on the remaining panels. The corrected caption of **Figure 4** appears below.

“Region analysis shows the percentage of NPA-specific germinal center B cells on days 10 (B), 20 (C) and 35 (A) in the spleens of mice that had been inoculated with NPA-liposomes. Percentage of NPA-specific germinal center B cells on day 35 in the spleens of mice inoculated with saline solution (D) or with smooth liposomes (E). Percentages of germinal center B cells specific to NPA in the draining lymph nodes (F) and spleen (G).”

We used the same image to show the gating strategy (panel 4A) and the experimental result (panel 4D). The paragraph with the mistake appears below:

“Germinal center B cells specific for NPA (IgD^-^, CD19^+^, PNA^+^, NPA^+^) were identified by flow cytometry (**Figures 4A-D**) in the draining lymph nodes and the spleen of mice that received liposomes bearing NPA; these germinal center B cells were not found in mice that received only smooth liposomes or saline solution (**Figures 4E, F**). The percentage of NPA-specific germinal center B cells increased progressively, from 4.37% and 6.15% on day 10 to 16.37% and 19.87% on day 35 for the draining lymph nodes and the spleen, respectively (**Figures 4G, H**).”

Therefore, we deleted panel 4D, referred to panel 4A when describing the experimental result, and updated the letters on the remaining panels. A correction has been made to the section **Results** Section, subsection *NPA-specific germinal center B cells are found in mice with the lupus-like disease*, Paragraph 4:

“Germinal center B cells specific for NPA (IgD^-^, CD19^+^, PNA^+^, NPA^+^) were identified by flow cytometry (**Figures 4A**-**C**) in the draining lymph nodes and the spleen of mice that received liposomes bearing NPA; these germinal center B cells were not found in mice that received only smooth liposomes or saline solution (**Figures 4D**, **E**). The percentage of NPA-specific germinal center B cells increased progressively, from 4.37% and 6.15% on day 10 to 16.37% and 19.87% on day 35 for the draining lymph nodes and the spleen, respectively (**Figures 4F**, **G**).”

The original version of this article has been updated.

