# Supplementary figures and images for: Anti-Lipid IgG Antibodies Are Produced via Germinal Centers in a Murine Model Resembling Human Lupus
Source: Front Immunol. 2016 Sep 29;7:396. doi: 10.3389/fimmu.2016.00396 (PMC5040728; doi:10.3389/fimmu.2016.00396)

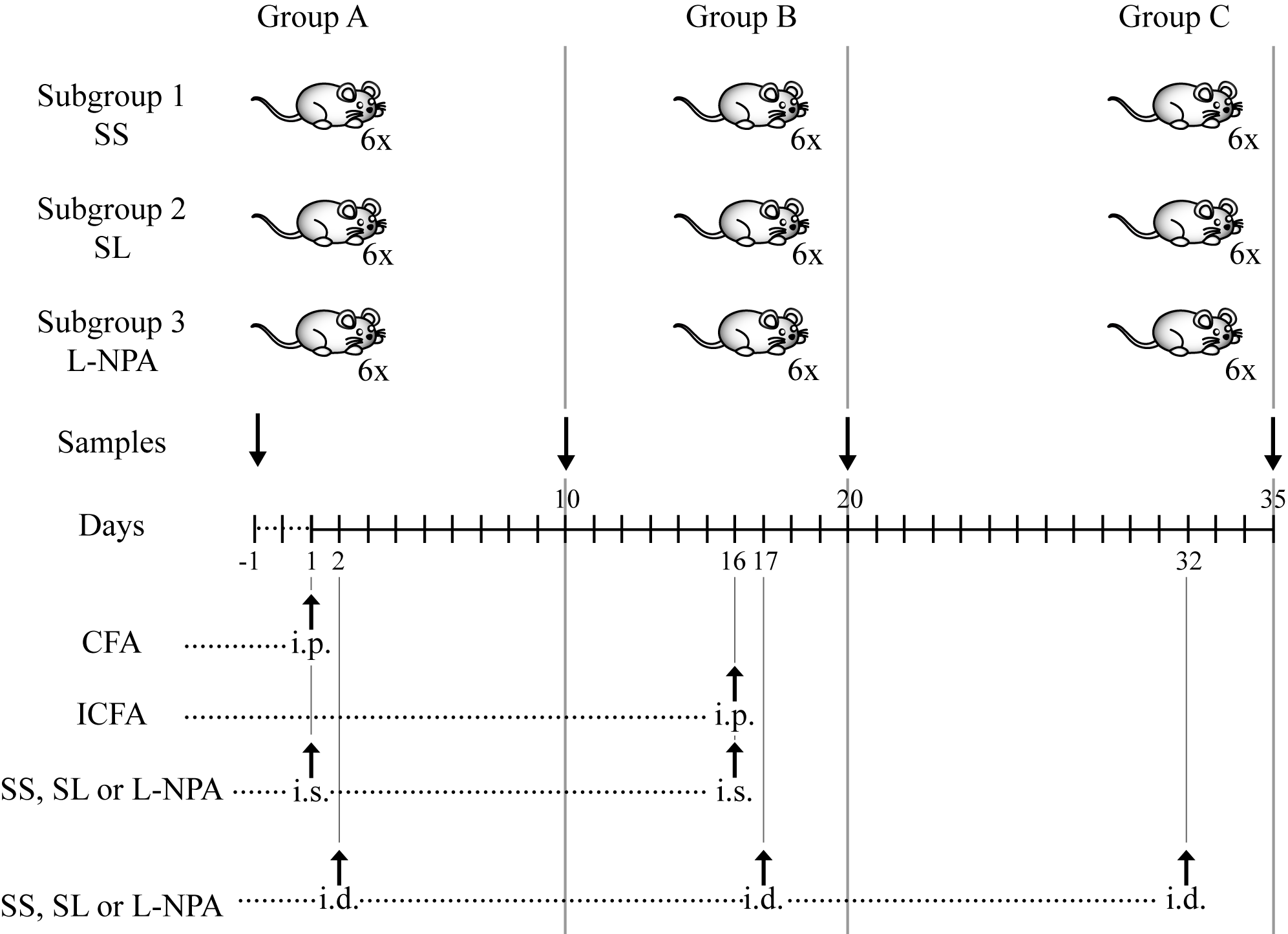

Supplement: Figure S1 — Three groups (A, B, and C) of adult mice were divided into three subgroups of six mice each. One subgroup received saline solution (SS), the other smooth liposomes (SL), and the last one liposomes bearing NPA (L-NPA). On day 1, group A received complete Freund’s adjuvant (CFA) (intraperitoneally, i.p.) and SS, SL, or L-NPA (intrasplenic, i.s.); on day 2, SS, SL, or L-NPA (intradermally, i.d.) and sacrificed on day 10. On day 16, group B received the same as group A plus incomplete Freund’s adjuvant (ICFA, i.p.) and SS, SL, or L-NPA (i.s.); on day 17, SS, SL, or L-NPA (i.d.) and sacrificed on day 20. Finally, group C received the same as groups A and B plus SS, SL, or L-NPA (i.d.) on day 32 and sacrificed on day 35. Samples were taken before immunization and on the days of sacrifice. [file Image_1.TIF]

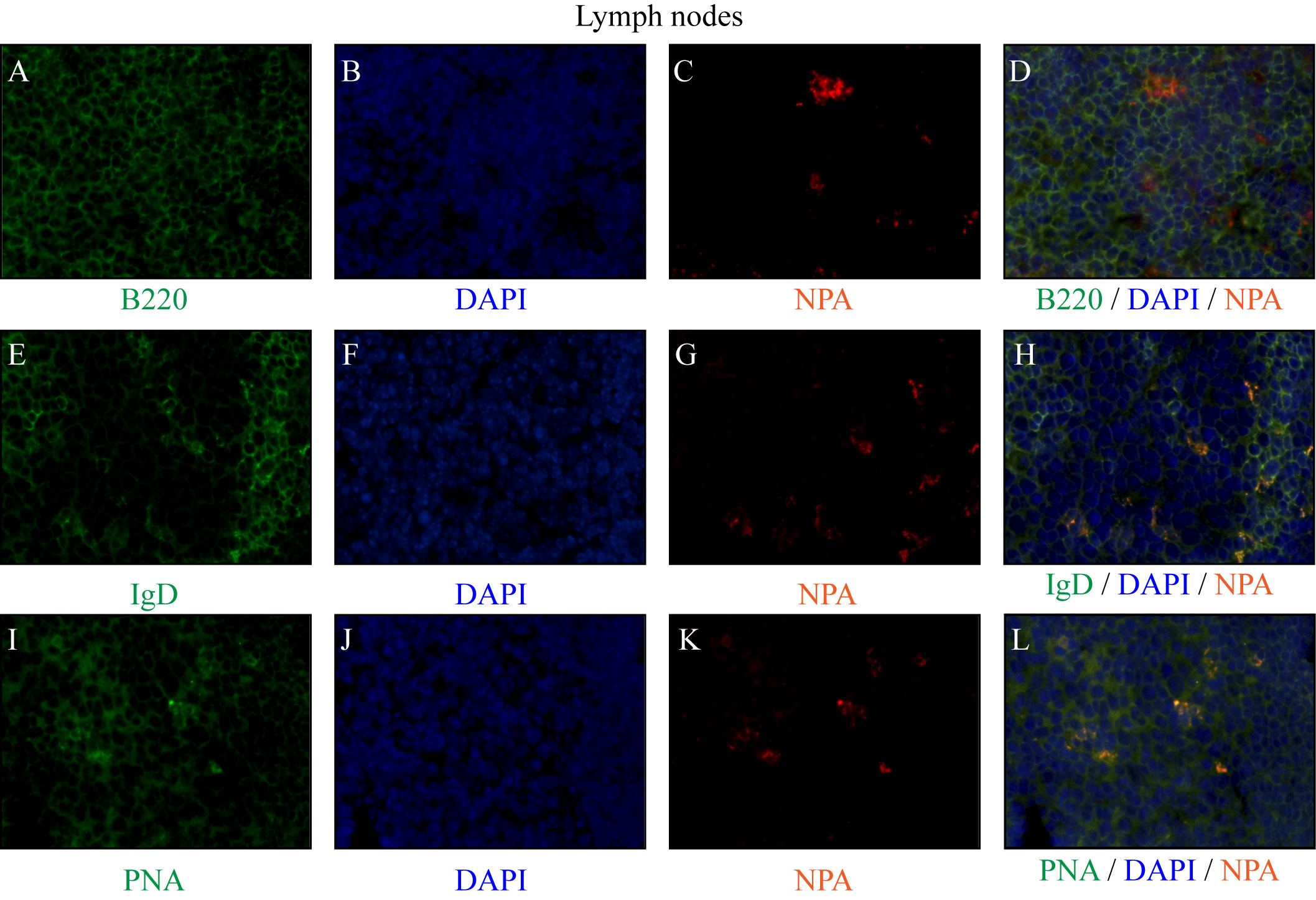

Supplement: Figure S2 — Individual and merged images of labeled tissue sections from draining lymph nodes. Individual images of the draining lymph nodes from mice with lupus-like disease induced by NPA-immunizations were taken with the Olympus BX51 microscope; green fluorescence for B220 (A), IgD (E), and PNA (I); blue fluorescence for nuclei counter-staining (DAPI) (B,F,J) and red fluorescence for NPAs (C,G,K). Merged images of B220/DAPI/PNA (D), IgD/DAPI/NPA (H), and PNA/DAPI/NPA (L). Images were merged with Image-Pro PLUS software. [file Image_2.TIF]
